# Coaxially Bioprinted Cell-Laden Tubular-Like Structure for Studying Glioma Angiogenesis

**DOI:** 10.3389/fbioe.2021.761861

**Published:** 2021-10-01

**Authors:** Xuanzhi Wang, Xinda Li, Yi Zhang, Xiaoyan Long, Haitao Zhang, Tao Xu, Chaoshi Niu

**Affiliations:** ^1^ Department of Neurosurgery, The First Affiliated Hospital of USTC, Division of Life Sciences and Medicine, University of Science and Technology of China, Hefei, China; ^2^ Department of Neurosurgery, Sichuan Provincial People’s Hospital, University of Electronic Science and Technology of China, Chengdu, China; ^3^ Chinese Academy of Sciences Sichuan Translational Medicine Research Hospital, Chengdu, China; ^4^ Tsinghua Shenzhen International Graduate School, Tsinghua University, Shenzhen, China; ^5^ East China Institute of Digital Medical Engineering, Shangrao, China; ^6^ Biomanufacturing and Rapid Forming Technology Key Laboratory of Beijing, Department of Mechanical Engineering, Tsinghua University, Beijing, China; ^7^ Department of Precision Medicine and Healthcare, Tsinghua Berkeley Shenzhen Institute, Shenzhen, China

**Keywords:** coaxial bioprinting, glioma, angiogenesis, fused cells, transdifferentiate

## Abstract

Glioblastomas are the most frequently diagnosed and one of the most lethal primary brain tumors, and one of their key features is a dysplastic vascular network. However, because the origin of the tumor blood vessels remains controversial, an optimal preclinical tumor model must be established to elucidate the tumor angiogenesis mechanism, especially the role of tumor cells themselves in angiogenesis. Therefore, shell-glioma cell (U118)-red fluorescent protein (RFP)/core-human umbilical vein endothelial cell (HUVEC)-green fluorescent protein (GFP) hydrogel microfibers were coaxially bioprinted. U118–RFP and HUVEC–GFP cells both exhibited good proliferation in a three-dimensional (3D) microenvironment. The secretability of both vascular endothelial growth factor A and basic fibroblast growth factor was remarkably enhanced when both types of cells were cocultured in 3D models. Moreover, U118 cells promoted the vascularization of the surrounding HUVECs by secreting vascular growth factors. More importantly, U118–HUVEC-fused cells were found in U118–RFP/HUVEC–GFP hydrogel microfibers. Most importantly, our results indicated that U118 cells can not only recruit the blood vessels of the surrounding host but also directly transdifferentiate into or fuse with endothelial cells to participate in tumor angiogenesis *in vivo*. The coaxially bioprinted U118–RFP/HUVEC–GFP hydrogel microfiber is a model suitable for mimicking the glioma microenvironment and for investigating tumor angiogenesis.

## Introduction

Glioblastomas (GBMs) are the most diagnosed primary malignant central-nervous-system tumors in adults and have a very poor prognosis. The five- and 2 years survival rates are only 4–5% and 26–33%, respectively. An important GBM characteristic is the abundance of abnormal vascular systems, and this uncontrolled vascular growth plays a crucial role in the occurrence, progression, and invasion of GBMs (Ostrom et al., 2018). Although some targeted therapies are available for tumor angiogenesis, they often exhibit drug resistance and limited efficacy because the molecular mechanism of tumor angiogenesis remains controversial (Ameratunga et al., 2018). Therefore, an ideal tumor angiogenesis model must be established to study the mechanism of tumor angiogenesis.

The traditional method of studying tumor angiogenesis *in vitro* mainly involves culturing tumor and endothelial cells in two-dimensional (2D) Petri dishes, while *in-vivo* studies mainly rely on animal models (Verbridge et al., 2010). Although 2D cultures are easily manipulated and culture conditions are controllable, cultured cells grow as a monolayer, lack cell–cell and cell–extracellular matrix interactions, and cannot mimic the three-dimensional (3D) structures of the tumor-tissue microenvironment *in vivo*. Furthermore, cellular interactions with the 3D microenvironment are crucial for tumor growth and angiogenesis (Wang et al., 2018). Moreover, 2D cultured cells have sufficient oxygen and nutrients, which is inconsistent with the hypoxic environment and concentration gradient of solid tumors *in vivo*. Consequently, 2D models can produce misleading results and provide false guidance for clinical trials. Additionally, cytokines secreted by 2D-cultured cells diffuse into the medium and cannot reach an effective biological concentration, which is not conducive to cellular paracrine and autocrine functions and is different from the protein expression, cell-signal transduction, cell activity, and drug response of tumor cells *in vivo* (Wang et al., 2018; Yi et al., 2019). Because of species differences between animal and human models, some experimental animals lack an immune response, and xenograft tumors grow faster than human ones. Hence, immature blood vessels inside xenograft tumors do not correspond with tumorigenic blood vessels inside human tumors, and the results of animal experiments cannot accurately predict therapeutic efficacy in humans (Bray and Werner, 2018). Therefore, over the past few decades, researchers have developed various 3D models for studying tumor angiogenesis (Wang et al., 2021).

Resink et al. used the multicellular tumor spheres (MCTSs) of undifferentiated melanoma cells (NA8) to construct a melanoma angiogenesis model *in vitro* and found that after cocultivation with the human microvascular endothelial cell line (HMEC-1), HMEC-1 invaded and formed an NA8–MCTS network structure (Ghosh et al., 2007). Chiew et al. cocultured endothelial and liver cancer cells (HepG2) in a 3D model to study the interaction between endothelial and tumor cells. The results showed that endothelial cells had differentiated into tubular network-like structures in the cocultured 3D spheres and were then enhanced or inhibited by adding angiogenic factors or inhibitors, respectively (Chiew et al., 2017). Chen et al. suspended dextran microspheres coated with human umbilical vein endothelial cells (HUVECs) in fibrin gel and implanted human glioma cells (U87) on the upper layer of the gel to construct a 3D angiogenesis model *in vitro*. The results showed that when cocultured with U87, which can secrete vascular endothelial growth factor (VEGF), HUVECs were induced to sprout and form longer tubule-like structures. Moreover, this effect was enhanced by adding exogenous VEGF (Chen et al., 2009). Poldervaart et al. used 3D bioprinting to construct sodium alginate/Matrigel^®^ scaffolds loaded with human endothelial progenitor cells (HEPCs) and added gelatin particles that slowly released VEGF in specific scaffold regions. The angiogenesis ability of the matrix–gel scaffold containing both HEPCs and VEGF was investigated by implanting the scaffolds into the skin of nude mice. The results showed remarkable angiogenesis in the vicinity of VEGF-containing gelatin particles, suggesting that the long-term presence of VEGF promoted the vascularization of the HEPC-loaded scaffolds (Poldervaart et al., 2014).

In summary, most current 3D models of tumor angiogenesis involve coculturing tumor and endothelial cells in a 3D microenvironment and using vascular growth factors (VGFs) secreted by tumor cells or adding exogenous VGFs to promote the sprouting or tubular formation of endothelial cells (Smith et al., 2015). In these models, tumor cells and/or VGFs is/are 1) necessary condition(s) for promoting endothelial cell vascularization, which also leads to some model defects. For example, adding exogenous VGFs to the 3D coculture microenvironment changes the inherent VGF concentration therein, which is not conducive to tumor- and endothelial-cell paracrine and autocrine functions (Chiew et al., 2017). Moreover, directly mixing tumor and endothelial cells in a 3D environment will likely cause contact inhibition of both types of cells during growth and does not conform to tumor-cell distribution *in vivo* (Lazzari et al., 2018). Having a better understanding of tumor-cell biological functions in 3D coculture systems is crucial for studying endothelial cell angiogenesis because tumor cells can participate in tumor angiogenesis not only directly but also by transdifferentiating into endothelial cells (Mei et al., 2017). More importantly, tumor cells can recruit and influence surrounding endothelial cells to participate in tumor neovascularization by secreting VGFs (Jhaveri et al., 2016). Therefore, constructing a model that can maximize the inherent biological properties of tumor cells is the key to studying tumor angiogenesis.

Coaxial extrusion bioprinting is a novel method of constructing 3D cellular microenvironments. Owing to the diversity of coaxial channels, linear structures containing various materials and cells can be manufactured simultaneously (Idaszek et al., 2019). The classic “shell–core” structure consists of a biomaterial-supported shell and a cell-filled core in which cells are in a 3D microenvironment, which is conducive to inherent cellular biological functions (Wang et al., 2018). Ozbolat et al. encapsulated human umbilical vein smooth muscle cells in sodium alginate by coaxial bioprinting, and the resulting printed hollow-core structure was used to mimic vascular lumina. The sodium-alginate-encapsulated cells exhibited good cellular proliferation during long-term culture *in vitro*. Furthermore, histological studies have demonstrated the deposition of smooth muscle matrix and collagen on and around the inner lumen surface (Zhang et al., 2015). For example, using coaxial multichannel extrusion, Zhang et al. constructed urothelial and vascular tissues containing human urothelial, bladder, and smooth muscle cells and HUVECs and found that coaxial bioprinted multilayered tubular structures provided adequate nutrients for cells, thus promoting the growth and proliferation of embedded cells (Pi et al., 2018). However, all these studies have mainly focused on utilizing coaxial printing to construct geometries that mimic vascular structures and have neglected the roles and functions of tumor cells in angiogenesis in coaxially printed tumor models, especially when tumor and endothelial cells are cocultured in a 3D microenvironment. In our previous studies, coaxial bioprinting was used to construct shell–core hydrogel microfibers containing glioma cells, which exhibited good cellular activity and proliferability in a 3D hydrogel microenvironment, and glioma-cell VEGFR2 expression was enhanced in the core (Wang et al., 2018). Therefore, we hypothesized that coaxially bioprinted glioma cells would affect the angiogenesis of cocultured endothelial cells and participate in tumor angiogenesis.

In this study, shell-U118-red fluorescent protein (RFP)/core-HUVEC-green fluorescent protein (GFP) hydrogel microfibers were fabricated by coaxial extrusion bioprinting. The proliferability and secretability of U118 cells and HUVECs were analyzed using 3D models. The effects of the U118 cells on the chemotaxis, migration, and formation of HUVEC tubule-like structures were observed *in vitro*. Moreover, core HUVECs were harvested on days 1 and 9 to evaluate angiogenesis-related gene and protein expressions. Furthermore, the U118-laden hydrogel microfibers were transplanted into the subcutaneous tissue of nude mice to analyze whether the glioma cells in the hydrogel–microfiber matrix recruited host vascular endothelial cells *in vivo*, to determine the compositions of new xenograft blood vessels, and to study the effects of the coaxially bioprinted glioma cells on the vascularization ability of cocultured endothelial cells and their role in tumor angiogenesis.

## Materials and Methods

### Cell Culture and Lentiviral Transfection

Human glioma cell line U118 and human umbilical vein endothelial cell (HUVEC, passages 3–4) were provided by the Shanghai Institute of Cell Biology, Chinese Academy of Science (Shanghai, China), respectively. Both of the cells were cultured in Dulbecco’s modified Eagle’s medium (DMEM, Gibco) supplemented with 10% fetal bovine serum (FBS, Gibco). U118 and HUVECs were transfected with lentivirus-mediated RFP and GFP (Shanghai Genechem Co.,Ltd., Shanghai, China) according to the manufacturer’s protocol, respectively. Briefly, 1 × 10^4^–3 × 10^4^ cells were added into each well of a 24-well plate and cultured for 16–24 h at 37°C until the cell confluence reached 30%. The optimal cell infection multiplicity was determined in pre-experiment. 2–3 μl infection solution with 1 × 10^8^ TU/ml virus was added into each well, and the medium was replaced with fresh medium after 12–16 h. The fluorescent protein expression was observed under a fluorescence microscope 72 h after transfection. Subsequently, 2–4 µg/ml puromycin was added. The screening medium was replaced every 2–3 days until the virus-free cells were killed by puromycin. The concentration of puromycin was reduced to 0.5–1 µg/ml, and the untransfected cells were continued to be screened. Flow cytometry was used to detect the proportion of RFP-positive and GFP-positive cells.

### Coaxial Bioprinting Shell-Core Hydrogel Microfibers

Sodium alginate powder (Sigma, A0682) was sterilized by gamma ray and dissolved in 0.9% sodium chloride solution (w/v) to obtain 2% sodium alginate solution (w/v). For the construction of shell-U118-RFP/core-HUVEC-GFP hydrogel microfibers, U118-RFP cells were resuspended in 2% sodium alginate solution as shell stream with a concentration of 1 × 10^6^/ml. The collagen solution with concentration of 2 mg/ml was prepared according to the instructions of collagen gelation procedure, and 2 × 10^5^/ml HUVEC-GFP suspension was mixed with collagen solution uniformly in equal volume to obtain a core stream with cell concentration of 1×10^5^/ml and final collagen concentration of 1 mg/ml. The printing device is mainly consisted of a sheath/core nozzle, which composed of two concentric circles (inner diameters:0.577 mm, 1.469 mm, respectively). For printing, the shell stream and core stream above were loaded into two 5 ml syringes, respectively. The extrusion speed of shell stream was set as 15 ml/h and core stream as 5 ml/h at a microinjection pump. Cell-laden hydrogel microfibers were obtained by crosslinking sodium alginate with 3% calcium chloride solution. Shell-U118-RFP/core-HUVEC-GFP hydrogel microfibers were maintained in DMEM medium supplemented with 10% FBS at 37°C, 5% CO_2_.

For control group, shell-U118-RFP/core hydrogel microfibers were prepared with no HUVEC-GFP in core stream, and shell/core-HUVEC-GFP hydrogel microfibers were prepared with no U118-RFP in shell stream.

### Biological Analysis of Cells in 3D Hydrogel Microfibers

Alamar Blue Kit (Shanghai, China) was used to detect cell proliferation on days 1, 3, 5, 7 and 9 of culture, respectively. Briefly, samples were immersed in working solution with 1800 μl fresh medium and 200 μl Alamar Blue and incubated for 2 h in dark at 37°C. Subsequently, 100 μL of supernatant was transferred to a 96-well plate and the optical density (OD) value was obtained at 570 and 630 nm wavelengths on a microplate reader. The OD value of each group was normalized to day 1 for statistic analysis. Similarly, VEGFA and bFGF secreted by cells in hydrogel microfibers were analyzed on days 1, 3, 5, 7 and 9 with a sandwich enzyme immunoassay kit (Donglin Sci&Tech, Wuxi, China) following the instructions. Briefly, 100 μl supernatant from hydrogel microfibers and standard solutions at different concentrations were added to well plates, respectively. After 2 h incubation, the solution from well plates was aspirated, and 100 μl detection reagent A was added for another 1 h culture. Then 100 μl detection reagent B was added for 1 h culture again. After that, 90 μl of substrate solution was added and incubated for 20min away from light. Finally, 50 μl of stop solution was added and the OD value was obtained at 450 nm wavelength. The standard curve was established according to the OD values of standard solution and the concentration in samples was calculated.

### *In vitro* Analysis of Vascularization Ability of Human Umbilical Vein Endothelial Cells-Green Fluorescent Protein

The morphological change, chemotactic migration and tubule-like structure formations of HUVEC-GFP in hydrogel microfibers were observed under an inverted fluorescence microscope on days 1, 5 and 9 of culture. In order to quantify the tubule-like structures, Image J software (Rawak Software, Inc., Germany) was used to analyze the number of tubules, which was defined as the closed loop formed by HUVEC-GFP. Briefly, the Angiogenesis Analyze tool and Network Analysis Menu of Image J software were operated. Here, the number of tubule-like structures was used to assess the vascularization ability of HUVEC-GFP (Donovan et al., 2001).

As described previously, Ca-alginate shell was dissolved with sodium citrate (Sigma Aldrich, Shanghai, China) to harvest HUVEC-GFP in the core at days 1 and 9, respectively (Wang et al., 2018). Quantitative real time PCR was used to evaluate the gene expression of CD31 and VEGFR2 in HUVEC-GFP. Briefly, cells were sufficiently dissociated by Trizol (Invitrogen, 15596–026) and total RNA was extracted according to the instructions. ImProm-IITM Reverse Transcription System (Promega, A3800) was used to reverse transcribe mRNA into cDNA. DNA transcription was performed using SYBR Green qPCR Super Mix and GAPDH was used as an internal standard. Relative gene expression was calculated using the 2^−ΔΔCt^ method. Western blot was performed to analyze the protein expression of CD31 and VEGFR2 in HUVEC-GFP. Briefly, cells were harvested and lysed with RIPA buffer (KeyGEN BioTECH, Nanjing, China). BCA protein assay kit (KeyGEN BioTECH, Nanjing, China) was used to evaluate the total protein concentration, and transferred to Immobilon-PPVDF membranes (Millipore, CT, United States). Then the protein was blocked using 5% skim milk solution for 1 h and incubated overnight at 4°C with primary antibodies (anti-VEGFR2 (ab134191), anti-CD31 (ab9498), all form abcam). GAPDH was used as the internal reference. The gray value of protein bands was evaluated by Image J software, and the level of target protein was normalized to the internal reference for plotting and statistics.

### Establishment of Subcutaneous Xenotransplanted Tumor Model

The design and implementation of all animal experiments were approved by the Institutional Ethical Board of the First Affiliated Hospital of USTC. BALB/c nude mice (4–6 weeks old) were anesthetized by intraperitoneal injection of 1% pentobarbital sodium solution (30 mg/kg). The dorsal skin was cut under sterile condition, and subcutaneous tissue was dissociated. The shell-U118-RFP hydrogel microfibers, which were cultured *in vitro* for 7 days were transplanted into the subcutaneous tissue of nude mice, and then the skin incision was sutured. After the operation, the nude mice were kept in separate cages and the incision was disinfected regularly.

### Histological Analysis of Xenograft Tumors

All subcutaneous xenograft tumors were obtained at 6 weeks after transplantation and fixed with 4% paraformaldehyde at 4°C overnight. Samples were stained with hematoxylin and eosin according to the instructions to analyze the presence of hydrogel and glioma cells within xenograft tumors. Immunohistochemical staining was performed with primary antibodies (anti-human/mouse CD31 (ab28364), anti-human CD105 (ab114052), anti-human/mouse CD105 (ab107595), all form abcam) following the manufacturer’s instructions. Immunofluorescence staining was carried out using primary antibodies (rabbit anti-human vWF (ab154193) form abcam, mouse anti-human GFAP (MAB2594) form R&D Biosystems) according to the protocol. In this study, CD31 was used to detect the neovascularization within xenograft tumors and CD105 was used to evaluate the composition of neovascularization. Particularly, vWF/GFAP double immunofluorescence staining was used to evaluate the origin of neovascularization.

### Analysis of Microvessel Density

MVD was determined by CD105 immunohistochemical staining as previously described (Weidner, 1995). Briefly, the areas with most abundant neovascularization were found under low power fields (40×magnification).The images were captured under 200 ×fields (Olympus IX51 microscope, 0.74 mm^2^ per field).The number of microvessels was calculated with Image J software. Any brown-stained endothelial cells or clusters of endothelial cells were considered to be a single, countable microvessel. Five different fields of CD105 positive cells or cell clusters were evaluated. MVD was defined as the number of microvessels calculated under a 200×field of view (0.74 mm^2^).

### Statistical Analysis

Data were presented as mean ± standard deviation and the results were analyzed by GraphPad Prism 7 software. The Student’s t-test was used to compare means between two groups. Comparisons between multiple groups were performed using two-way analysis of variance and a Bonferroni post-hoc test. *p < 0.05, **p < 0.01 was considered as statistically significant.

## Results

### Construction of U118–Red Fluorescent Protein and Human Umbilical Vein Endothelial Cells-Green Fluorescent Protein Cells

**FIGURE 1:**
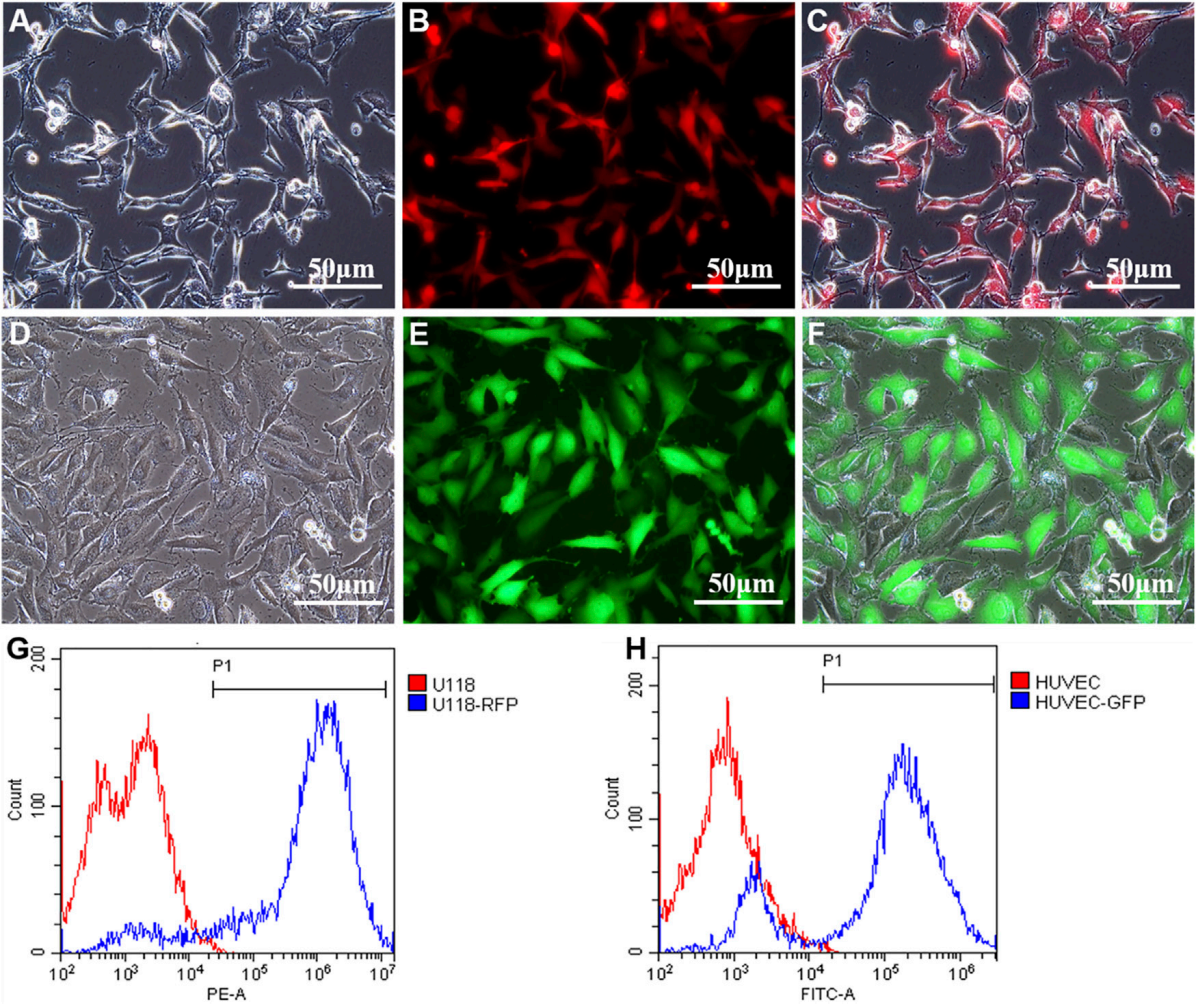
Construction of U118-RFP and HUVEC-GFP. **(A-C)** U118 cells were transfected with RFP. **(D-F)** HUVEC cells were transfected with GFP. **(G)** Proportion of RFP-positive cells. **(H)** Proportion of GFP-positive cells.

To better observe the effects of U118 cells on the morphological structure of cocultured HUVECs in a coaxially printed tumor model, U118 cells and HUVECs were first transfected with RFP and GFP, respectively. As shown in Figures 1A–F, U118–RFP and HUVEC–GFP cells were well established by lentiviral transfection. After 10 days of transfection and screening, flow cytometry was used to detect the transfection efficiency. Figures 1G,H shows that the proportions of RFP- and GFP-positive cells were 96.67 ± 2.15 and 85.73 ± 4.68%, respectively.

### Coaxially Bioprinted Glioma Microenvironment

**FIGURE 2:**
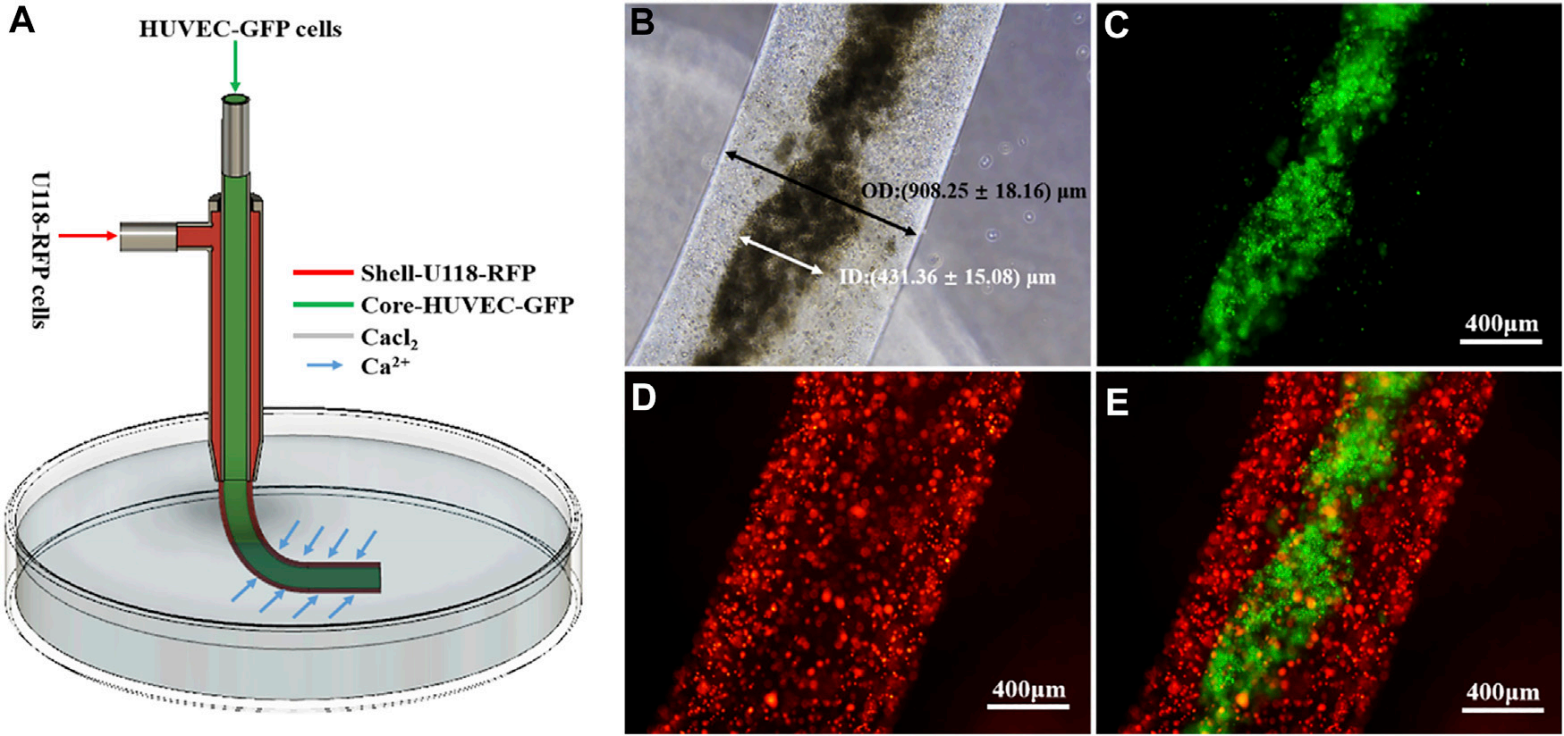
Coaxially bioprinted glioma microenvironment. **(A)** Schematic showing fabrication of cell-laden shell–core hydrogel microfibers. **(B-E)** Shell-U118–RFP/core-HUVEC–GFP hydrogel microfiber.

Figure 2A shows a schematic illustrating the construction of cell-laden shell–core hydrogel microfibers. As shown in Figure 2B, the inner and outer diameters of the microfibers were 431.36 ± 15.08 and 908.25 ± 18.16 µm, respectively. Figures 2C–E shows that a Ca–Na alginate shell loads and encapsulates core U118–RFP and HUVEC–GFP cells, respectively, which together constitute the glioma microenvironment (i.e., shell-U118–RFP/core-HUVEC–GFP hydrogel microfibers).

## Proliferability and Secretability of Cells in 3D Hydrogel Microfibers

**FIGURE 3:**
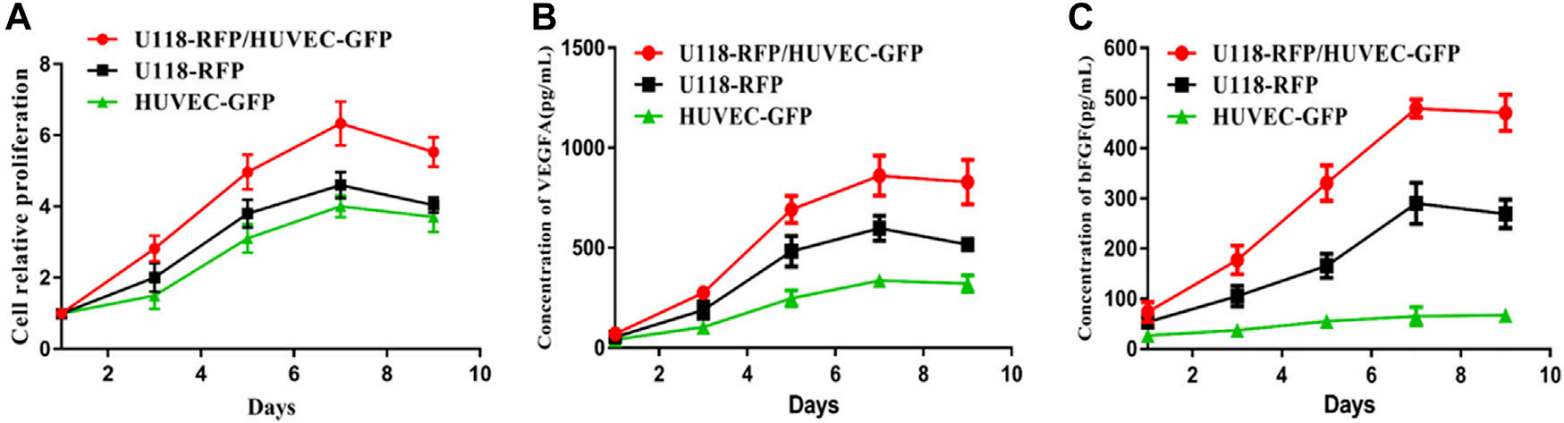
Proliferability and secretability of cells in 3D hydrogel microfibers. **(A)** Proliferation of U118–RFP and HUVEC–GFP in 3D hydrogel microenvironment. **(B)** Concentration of VEGFA secreted by U118–RFP and HUVEC–GFP cells. **(C)** Concentration of bFGF secreted by U118–RFP and HUVEC–GFP cells.

As shown in Figure 3A, U118–RFP and HUVEC–GFP both exhibited good cellular proliferation in the 3D hydrogel microenvironment. Moreover, when both types of cells were cocultured in hydrogel microfibers, both exhibited satisfactory cellular proliferation. Furthermore, Figure 3B shows that both U118–RFP and HUVEC–GFP secreted VEGFA and that the VEGFA secretability of both types of cocultured cells was remarkably more pronounced than that of either type of cell cultured individually. As shown in Figure 3C, although HUVEC–GFP negligibly secreted bFGF, its bFGF secretability was remarkably enhanced when U118–RFP and HUVEC–GFP were cocultured in hydrogel microfibers.

### Vascularization Ability of Human Umbilical Vein Endothelial Cells-Green Fluorescent Protein in 3D Hydrogel Microfibers

**FIGURE 4:**
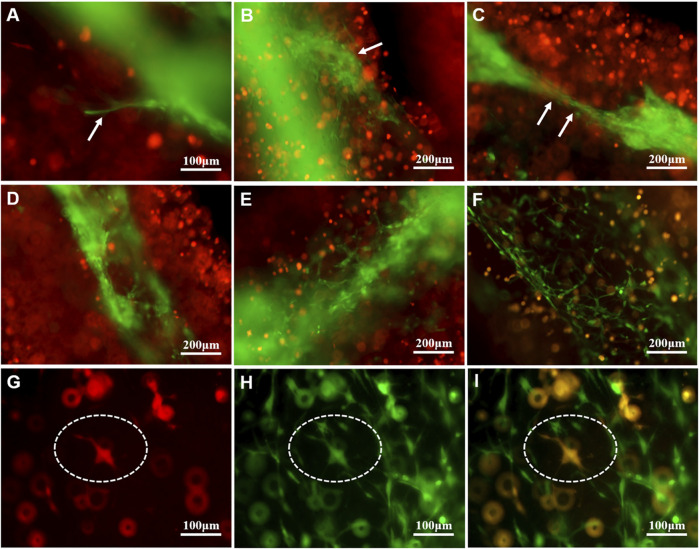
Morphological changes of HUVEC–GFP in U118–RFP/HUVEC–GFP hydrogel microfiber. **(A-C )** HUVEC–GFP core exhibits chemotaxis and migration (as indicated by white arrows). **(D-F)** HUVEC–GFP gradually formed cobblestone-like structure. **(G-I)** Cultured U118–RFP^+^/HUVEC–GFP^+^-fused cells at 9 days (as indicated by dotted ovals).

**FIGURE 5:**
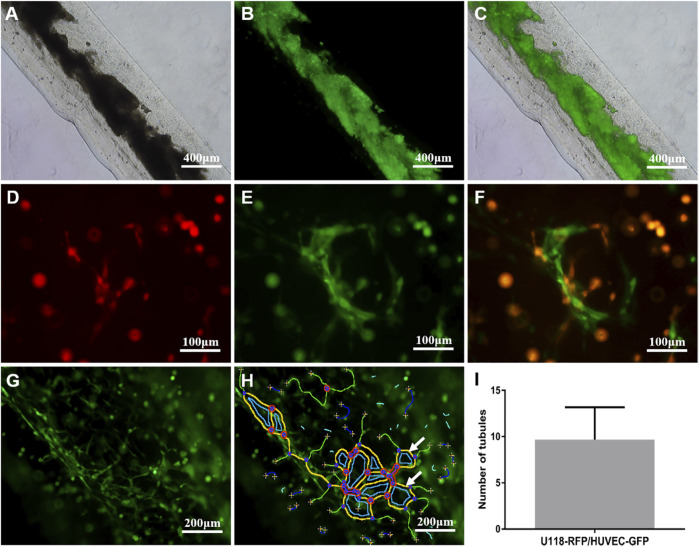
Tubule-like structures formed by HUVEC–GFP. **(A-C)** HUVEC–GFP did not form tubular-like structures in HUVEC–GFP hydrogel microfiber. **(D-F)** HUVEC–GFP formed tubular-like structures in U118–RFP/HUVEC–GFP hydrogel microfiber. **(G-I)** Tubule-like structures were analyzed using ImageJ software (indicated by white arrows).

As shown in Figure 4A, on the first day of the U118–RFP/HUVEC–GFP hydrogel microfiber culture, the core HUVEC–GFP appeared to sprout. After 5 days, the core HUVEC–GFP exhibited chemotaxis and began migrating to the U118–RFP-cell-laden shell (Figure 4B). Moreover, HUVEC–GFP cells began connecting and bridging gaps between cells (Figure 4C). Figures 4D–F shows that HUVEC–GFP gradually formed cobblestone-like structures on days 1, 5, and 9. Notably, U118–RFP^+^/HUVEC–GFP^+^-fused cells appeared after 9 days (Figures 4G–I). Interestingly, although no HUVEC–GFP-induced tubule-like structures had formed in the HUVEC–GFP hydrogel microfibers after 9 days (Figures 5A–C), the HUVEC–GFP in the U118–RFP/HUVEC–GFP hydrogel microfibers formed 9.67 ± 3.51 tubules (Figures 5D–F and Figures 5G–I).

**FIGURE 6:**
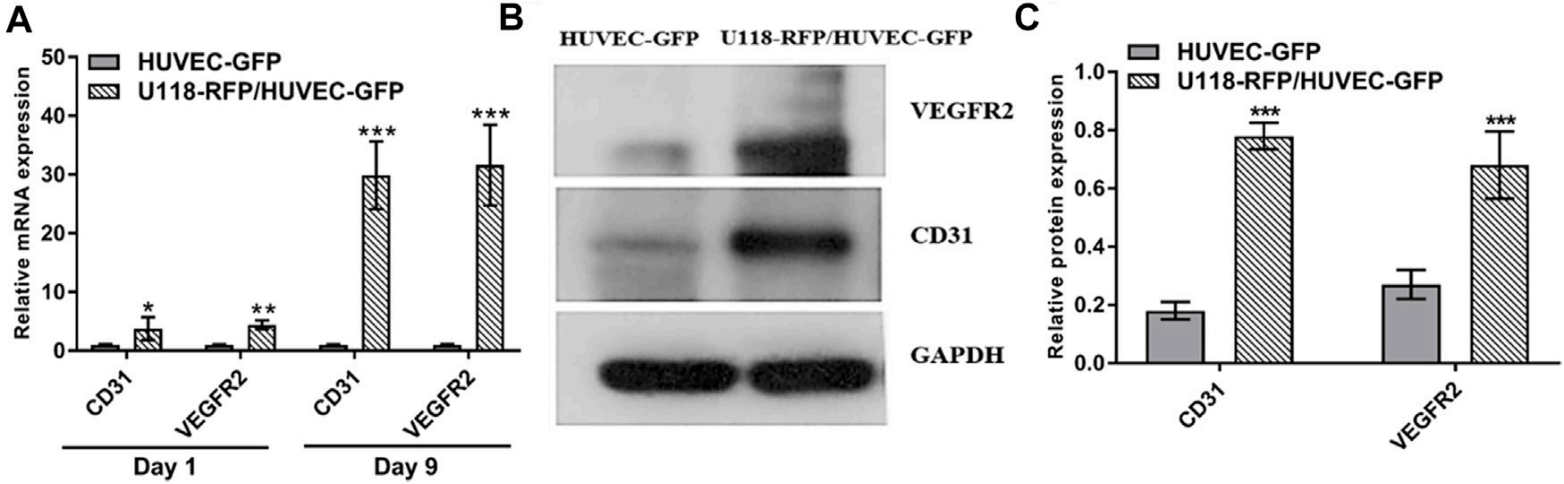
Expression of CD31 and VEGFR2 in HUVEC–GFP. **(A)** Relative mRNA expressions of CD31 and VEGFR2 in U118–RFP/HUVEC–GFP hydrogel microfiber were both higher than those in HUVEC–GFP hydrogel microfiber on days 1 and 9. **(B, C)** Protein expressions of CD31 and VEGFR2 in U118–RFP/HUVEC–GFP hydrogel microfiber were both higher than those in HUVEC–GFP hydrogel microfiber at day 9.

To evaluate angiogenesis-related gene expression, real-time quantitative reverse transcription polymerase chain reaction (i.e., qRT–PCR) was used to determine the mRNA expressions of CD31 and VEGFR2 in HUVECs. As shown in Figure 6A, the relative mRNA expression of CD31 in the U118–RFP/HUVEC–GFP hydrogel microfibers was 3.78 ± 1.94- and 29.88 ± 5.78-fold higher than that in the HUVEC–GFP hydrogel microfibers on days 1 and 9, respectively. Moreover, the relative mRNA expression of VEGFR2 in the U118–RFP/HUVEC–GFP hydrogel microfibers increased to 4.44 ± 0.77 -and 31.64 ± 6.85-fold higher than that in the HUVEC–GFP hydrogel microfibers on days 1 and 9, respectively. In addition, western blotting was used to evaluate the protein expressions of CD31 and VEGFR2 in HUVEC–GFP on day 9. As shown in Figures 6B,C, the protein expressions of CD31 and VEGFR2 were significantly higher in the U118–RFP/HUVEC–GFP hydrogel microfibers than in the HUVEC–GFP ones on day 9.

### Neovascularization of U118–Red Fluorescent Protein Xenograft Tumor

**FIGURE 7:**
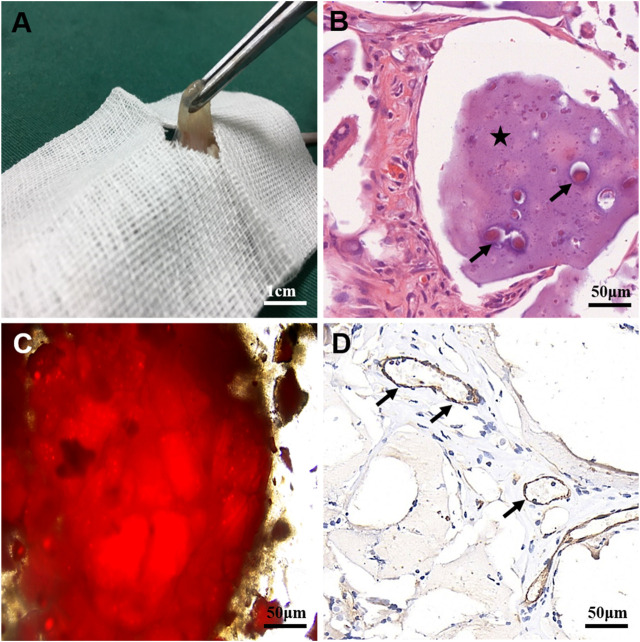
Characteristics of U118–RFP xenograft tumor. **(A)** Xenograft tumor exhibits soft texture and “fish-like” color change. **(B)** Residual hydrogel in tumor (as indicated by asterisk) containing U118–RFP cells (as indicated by black arrow). **(C)** U118–RFP cells in xenograft tumor (as indicated by red fluorescent protein). **(D)** Expression of CD31 in xenograft tumor (as indicated by black arrows).

**FIGURE 8:**
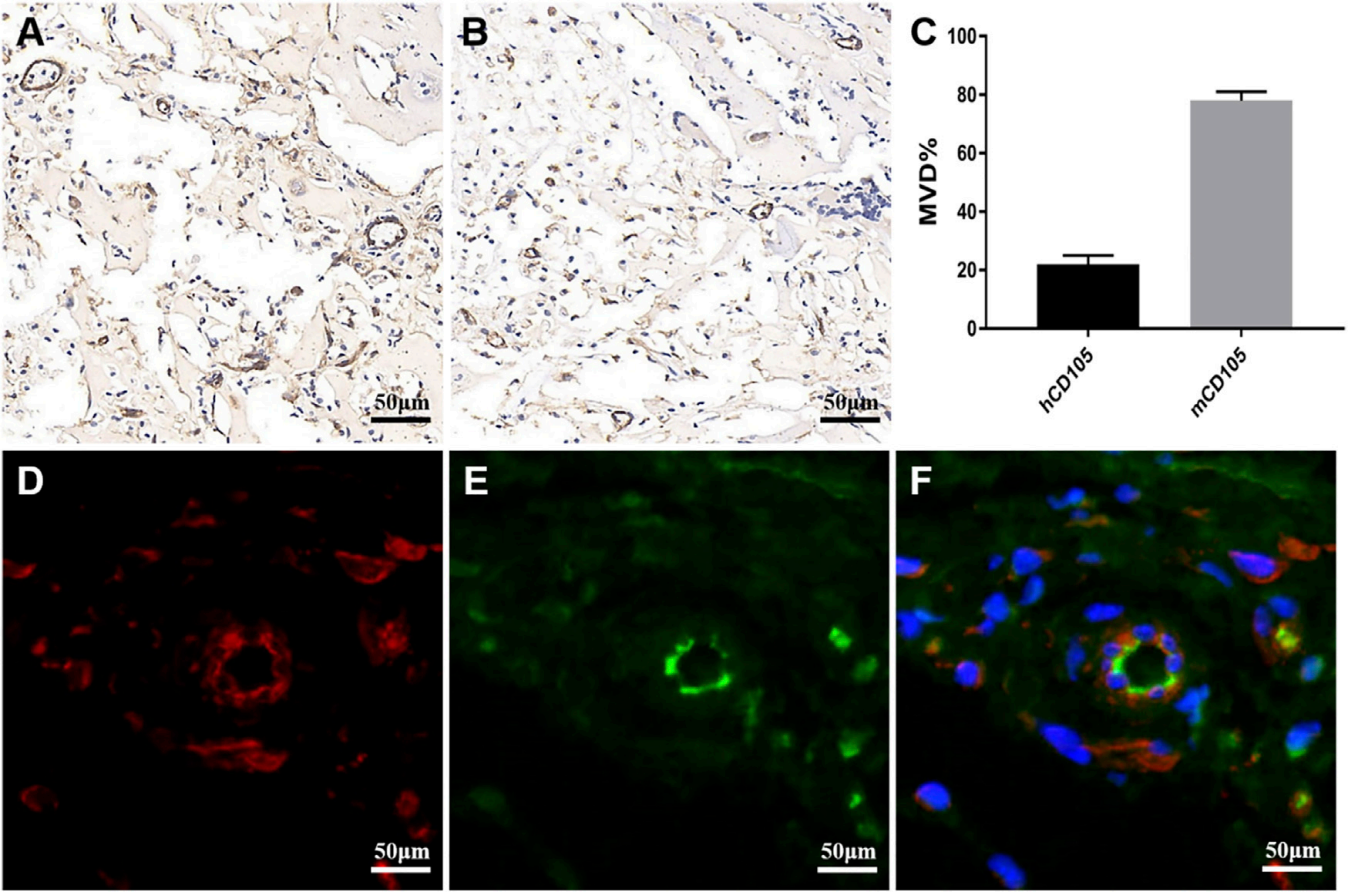
Neovascularization composition in xenograft tumor. **(A)** Immunohistochemical staining of xenograft tumor labeled with antihuman/murine CD105. **(B)** Immunohistochemical staining of xenograft tumor labeled with antihuman CD105. **(C)** MVD of human (h) and mouse (m) CD105^+^cells in xenograft tumor. **(D-F)** Xenograft tumor was labeled with antihuman vWF (green) and antihuman GFAP (red) by double immunofluorescence staining. Nuclei are counterstained blue.

To analyze whether glioma cells recruited host vascular endothelial cells in hydrogel microfibers *in vivo* and the origin and composition of new xenograft blood vessels, U118–RFP xenograft tumors were constructed. As shown in Figure 7A, xenograft tumors exhibited a soft texture and “fish-like” color change very similar to the morphology of human intracranial gliomas. Figures 7B,C, shows residual hydrogel inside the tumor and U118–RFP cells scattered in the hydrogel. Specifically, immunohistochemical staining of CD31 demonstrated neovascularization in xenograft tumors (Figure 7D). As shown in Figures 8A,B, the neovascularization inside the xenograft tumor contained both human endothelial-like cells labeled with human-specific antiCD105 and murine endothelial-like cells labeled with antihuman/mouse CD105. Notably, 78 and 22% of the CD105^+^ cells in tumors were murine and human, respectively (Figure 8C). The human endothelial-like cells in the U118–RFP-derived tumors were confirmed by costaining xenogaft tumors with antihuman von Willebrand factor (vWF) and glial fibrillary acidic protein (GFAP) antibodies. Figures 8D–F shows that a proportion of endothelial cells coexpressed both GFAP and vWF.

## Discussion

An important characteristic of GBM is the abundance of abnormal vascular systems, and this uncontrolled vascular growth plays a crucial role in the occurrence, progression, and invasion of tumor (Carlson et al., 2020). Elucidating and understanding the molecular mechanism of tumor angiogenesis is very important for the targeted therapy of antitumor angiogenesis. Therefore, an ideal tumor angiogenesis model must be established to study the mechanism of tumor angiogenesis.

In this study, cell-laden shell–core hydrogel microfibers were manufactured using coaxial bioprinting. To observe the interaction between tumor and endothelial cells in the shell–core hydrogel microfiber more intuitively, U118–RFP cells and HUVEC–GFP cells were constructed. The proportions of RFP-positive and GFP-positive cells were 96.67 ± 2.15 and 85.73 ± 4.68%, respectively. Good transfection efficiency helps to better distinguish and observe morphological changes between both cell types. In this study, Ca–Na alginate as a shell structure loaded U118–RFP cells and encapsulated HUVEC–GFP cells in the core, which together constitute the glioma microenvironment (shell-U118–RFP/core-HUVEC–GFP hydrogel microfibers). With this model, it is helpful to investigate the vascularization effect of tumor cells on endothelial cells in a 3D microenvironment.

Good cell proliferation after bioprinting is the premise of biological efficacy. Both U118–RFP and HUVEC–GFP cells showed good cell proliferation activity in hydrogel microfibers. In particular, when the two types of cells were cocultured in a 3D hydrogel microenvironment, they showed satisfactory proliferation ability and reached the maximum on day 7. As described previously, VEGF promotes angiogenesis and increases vascular permeability, which plays a pivotal role in tumor angiogenesis (Srivastava et al., 2020). In particular, VEGFA is a major player in physiological and tumor-induced angiogenesis, and numerous human tumors, including GBM, have shown VEGFA overexpression (Andreozzi et al., 2014). In this study, U118–RFP and HUVEC–GFP both secreted VEGFA. More importantly, the VEGFA secretability of both types of cells cocultured in a microenvironment was remarkably stronger than that of either of them. In addition, we evaluated bFGF, another factor that can induce angiogenesis (Chen et al., 2021). Our results showed that although U118–RFP and HUVEC–GFP weakly secreted bFGF, their bFGF secretability was remarkably enhanced when both types of cells were cocultured in hydrogel microfibers possibly because when tumor and endothelial cells were cocultured in a 3D microenvironment, the paracrine and autocrine pathways of the cells stimulated the ability of both cells to secrete vascular growth factors (Zhou et al., 2021).

Several studies have demonstrated that vascular growth factors play an important role in angiogenesis (Domingues et al., 2021). As mentioned above, the 3D microenvironment composed of shell-U118–RFP/core-HUVEC–GFP was enriched in vascular growth factors. Here, we found that with increasing culture time, for U118–RFP/HUVEC–GFP hydrogel microfiber, core-HUVEC–GFP cells sprouted, chemotaxis, and migration to shell-U118–RFP cells; and finally, HUVEC–GFP cells connected with each other to form tubule-like structures. Interestingly, no tubule-like structures were observed in HUVEC–GFP hydrogel microfibers possibly because tumor cells had secreted vascular growth factor, which induced endothelial cell vascularization. Furthermore, fusion cells (U118–RFP^+^/HUVEC–GFP^+^ cells) were observed in the U118–RFP/HUVEC–GFP hydrogel microfiber culture on day 9. In our previous study, cancer and mesenchymal stem cells fused, which contributed to glioma angiogenesis (Sun et al., 2019; Dai et al., 2017). Moreover, growing evidence suggests that glioma stem cells participate in glioma angiogenesis by directly transdifferentiating into endothelial cells (Ricci-Vitiani et al., 2010). However, the fusion of tumor and vascular endothelial cells during angiogenesis has rarely been reported. Our results suggest that the fusion of a few tumor and endothelial cells may play a role in tumor angiogenesis.

To better evaluate the vascularization ability of core-HUVEC–GFP cells, CD31 and VEGFR2 were selected to analyze their expression levels in different 3D microenvironments. The VEGFR2-mediated signaling pathway plays an important role in the autocrine process of VEGF ligand, and the binding of VEGFA and VEGFR2 is the main factor involved in the regulation of angiogenesis (Abhinand et al., 2016). In this study, the gene and protein expression of VEGFR2 in U118–RFP/HUVEC–GFP hydrogel microfibers was significantly higher than that in HUVECs. CD31 is a marker used to evaluate tumor angiogenesis. Our results indicated that the protein and gene expression of CD31 in U118–RFP/HUVEC–GFP hydrogel microfibers was significantly higher than that in HUVECs. We speculated that the VEGFA and bFGF secreted by U118–RFP promoted VEGFR2 and CD31 expression through paracrine or autocrine pathways, and further enhanced its vascularization, especially when U118–RFP cells and HUVEC–GFP cells were cocultured in a 3D microenvironment.

To further investigate how U118–RFP cells participated in tumor angiogenesis *in vivo*, a U118–RFP xenograft tumor was established. The texture and color of xenograft tumors were both very similar to those of human intracranial gliomas. Moreover, because U118 cells carried RFP, xenograft tumors clearly were mainly composed of tumor cells and residual hydrogel. This suggested that the animal tumor model established using the U118–RFP hydrogel microfiber was successful. CD31 immunohistochemical staining demonstrated neovascularization within the xenograft tumors. Furthermore, human-specific antiCD105 and antihuman/mouse CD105 were used to investigate the vascular composition and origin of xenograft tumors. Our results indicated that neovascularization within the xenograft tumor contained both human and murine endothelial-like cells. Notably, 78 and 22% of the tumor CD105^+^ cells were murine and human, respectively, indicating that tumor cells can not only recruit the blood vessels of the surrounding host to participate in tumor angiogenesis but also directly participate in angiogenesis themselves. To further identify human U118–derived endothelioid cells, xenograft tumors were costained with antihuman vWF and antihuman GFAP antibodies. Our results showed that xenograft tumors contained a proportion of the tubule-like structures composed of endothelial/glial phenotypic (vWF^+^/GFAP^+^) cells, suggesting that U118 cells could directly transdifferentiate into or fuse with endothelial cells to participate in tumor angiogenesis. Tumor cells play a crucial role in tumor angiogenesis.

## Conclusion

Shell-U118–RFP/core-HUVEC–GFP hydrogel microfibers were fabricated using coaxial extrusion bioprinting. This model shows great potential in mimicking the glioma microenvironment that can be used to evaluate glioma angiogenesis. U118–RFP and HUVEC–GFP cells both showed good cellular proliferation in the 3D hydrogel microenvironment. VEGFA and bFGF secretabilities were both remarkably enhanced when both cell types were cocultured in the hydrogel microfibers. Moreover, U118 cells promoted the vascularization of the surrounding HUVECs by secreting vascular growth factors. More importantly, U118 and HUVEC fused cells were found in U118–RFP/HUVEC–GFP hydrogel microfibers. Our results indicated that U118 cells can not only recruit the blood vessels of the surrounding host to participate in tumor angiogenesis *in vivo* but also directly transdifferentiate into or fuse with endothelial cells to participate in tumor angiogenesis.

## Data Availability

The original contributions presented in the study are included in the article/Supplementary Material, further inquiries can be directed to the corresponding authors.
